# Madecassoside Inhibits Melanin Synthesis by Blocking Ultraviolet-Induced Inflammation

**DOI:** 10.3390/molecules181215724

**Published:** 2013-12-16

**Authors:** Eunsun Jung, Jung-A Lee, Seoungwoo Shin, Kyung-Baeg Roh, Jang-Hyun Kim, Deokhoon Park

**Affiliations:** 1 Biospectrum Life Science Institute, Eines Platz 11th FL, 442-13 Sangdaewon Dong, Seongnam City, 462-807 Gyunggi Do, Korea; E-Mail: bioso@biospectrum.com (E.J.); biofk@biospectrum.com (J.-A.L.); biost@biospectrum.com (S.S.); biosh@biospectrum.com (K.-B.R.); 2 Dermiskin Life Science Institute, 44-9 Cheongho Ri, Pyeongtaek City, 451-862 Gyunggi Do, Korea; E-Mail: kjh@dermiskin.com

**Keywords:** post-inflammatory pigmentation, madecassoside, PAR-2, UVR

## Abstract

Madecassoside (MA), a pentacyclic triterpene isolated from *Centella asitica* (L.), is used as a therapeutic agent in wound healing and also as an anti-inflammatory and anti-aging agent. However, the involvement of MA in skin-pigmentation has not been reported. This study was conducted to investigate the effects of MA on ultraviolet (UV)-induced melanogenesis and mechanisms in a co-culture system of keratinocytes and melanocytes. MA significantly inhibited UVR-induced melanin synthesis and melanosome transfer in the co-culture system. These effects were further demonstrated by the MA-induced inhibition of protease-activated receptor-2 expression and its signaling pathway, cyclooxygenase-2, prostaglandin E2 and prostaglandin F2 alpha in keratinocytes. The clinical efficacy of MA was confirmed on artificially tanned human skin. MA significantly reduced UV-induced melanin index at 8 weeks after topical application. Overall, the study demonstrated significant benefits of MA use in the inhibition of hyperpigmentation caused by UV irradiation.

## 1. Introduction

In mammals, pigmentation results from the synthesis and distribution of melanin in the skin, hair bulbs, and eyes. Hyperpigmentation is a common and distressing problem caused by various inflammatory skin disorders, such as eczema, allergic contact dermatitis, and irritant contact dermatitis [1,2]. Ultraviolet (UV) radiation indirectly influences the melanogenesis of melanocytes through a paracrine regulation process involving keratinocytes. UV stimulated the secretion of stem cell factor (SCF), α-melanocyte stimulating hormone (α-MSH), endothelin (ET-1), and adrenocorticotropin melanocyte stimulating hormone (ACTH) from keratinocytes and induces melanogenesis of melanocytes [1]. In addition, prostaglandin (PG) is a lipid signaling intermediate produced by cyclooxygenation of arachidonic acid through the action of cyclooxygenase (COX) enzymes and prostaglandin E2 (PGE_2_) synthase enzymes. In the epidermis, PGE_2_ and prostaglandin F2α (PGF_2_α) are the main PGs produced by keratinocytes in response to UV irradiation. Several reports have suggested that PGs mediate postinflammatory pigmentary changes by modulating melanin synthesis and melanocyte dendricity [3,4,5]. Protease-activated receptor (PAR)-2 is a member of a novel G-protein-coupled seven-transmembrane receptor family. In epidermis, PAR-2 is expressed in keratinocytes [6], but not melanocytes [7]. A central role for PAR-2 in keratinocyte uptake of melanosomes has been established [8,9]. PAR-2 has been linked to the upregulation of COX-2 and the release of arachidonic acid and secretion of PGE_2_ and PGF_2_α [10,11]. Several reports have suggested that PAR-2 mediates cutaneous pigmentation through increased uptake of melanosomes by keratinocytes and by the release of PGE_2_ and PGF_2_α that stimulate melanocyte dendricity [12].

**Figure 1:**
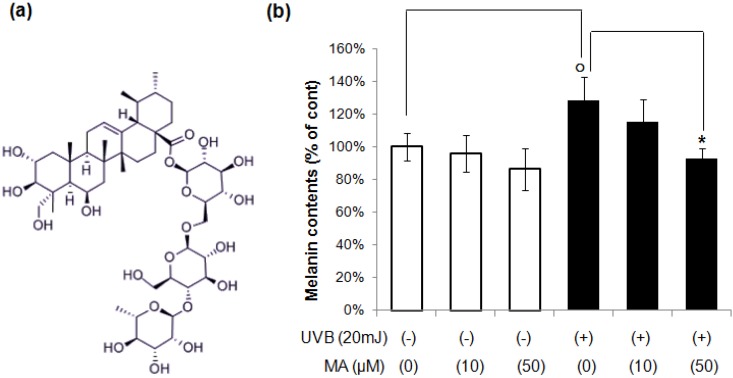
(**a**) Chemical structure of MA. (**b**) Melanocytes and HaCaT keratinocytes were co-cultured in a hanging cell culture insert system in the presence or absence of UVB irradiation (20 mJ/cm^2^) using 12-well Millicell Hanging Cell Culture Inserts. After HaCaT cells in upper chamber were irradiated with UVB, MA was added to the indicated concentration and then placed above the melanocytes. After 4 days, lower-chamber melanocytes were collected for melanin assay. The data shown are the means ± S.D., n = 3. ^o^* p* < 0.05 *vs.* UVB untreated control; * *p* < 0.05 *vs.* UVB-irradiated control; MA: Madecassoside.

Madecassoside (MA, Figure 1a), one of the major triterpene glycosides isolated from *Centella asiatica*, has various effects including wound healing [13], anti-inflammatory [14] and anti-aging activities [15,16], and protective effects against oxidative stress and UVB radiation [17,18]. The anti-inflammatory activity is due to inhibition of COX-2 and PG production [19,20]. However, the involvement of MA in skin-pigmentation has not been reported to date. In this study, we examined the the effects of MA on UVR-induced melanogenesis and its mechanisms of action in a co-culture system of keratinocytes and melanocytes.

## 2. Results and Discussion

### 2.1. Effect of MA on UVR-Induced Melanogenesis in Keratinocyte/Melanocytes Co-Cultures

To determine whether MA reduces UVB-induced melanin synthesis in the keratinocyte/melanocyte co-culture system, melanin contents were measured during co-culture. After HaCaT keratinocytes in the upper chamber were irradiated with UVB, MA were added to the indicated concentration and placed above the melanocytes. After 4 days, lower-chamber melanocytes were collected for assay of melanin. As shown in Figure 1b, the melanin content of melanocytes in co-culture with UVB-irradiated HaCaT keratinocytes was increased compared to co-culture with non-irradiated HaCaT keratinocytes. MA decreased the melanin content significantly in UVB-irradiated, co-cultured keratinocytes/melanocytes, whereas MA did not show any significant effect on melanin synthesis in non-irradiated, co-cultured keratinocytes/melanocytes. In addition, MA did not show any significant effects on melanin synthesis in single melanocyte model (supplementary data). These results suggest that MA inhibited melanin synthesis by blocking melanogenic stimulator released from keratinocytes by UVB irradiation. The results were verified by repeating the experiments three times, each of which was conducted in duplicate on melanocytes derived from the same donor.

### 2.2. Effect of MA on PGE_2_ and PGF_2_α Production in Keratinocytes

**Figure 2:**
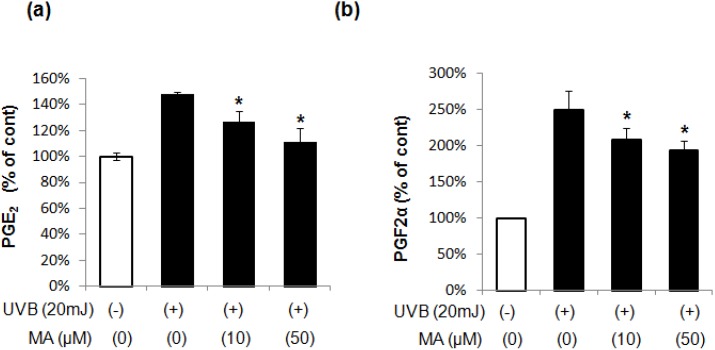
The levels of lipid mediators of inflammation (**a**) PGE_2_, (**b**) PGF_2_α. The data shown are the mean ± S.D., n = 3. * p < 0.05 *vs.* UVB-irradiated control; MA: Madecassoside.

PGE_2_ and PGF_2_α, which are the main PGs produced by keratinocytes in response to UV) irradiation, mediate postinflammatory pigmentation by modulating melanin synthesis and melanocyte dendricity. Therefore, we evaluated MA to determine its involvement in PGE_2_ and PGF_2_α production in UVB-irradiated keratinocytes. UVB irradiation markedly upregulated PGE_2_ and PGF_2_α. The upregulated production was suppressed by treatment with MA (Figure 2). These results suggest that MA inhibits UVB induced pigmentation by suppressing the production of PGE_2_ and PGF_2_α in keratinocytes.

### 2.3. Effect of MA on COX-2 and PAR-2 Expression in Keratinocytes

**Figure 3:**
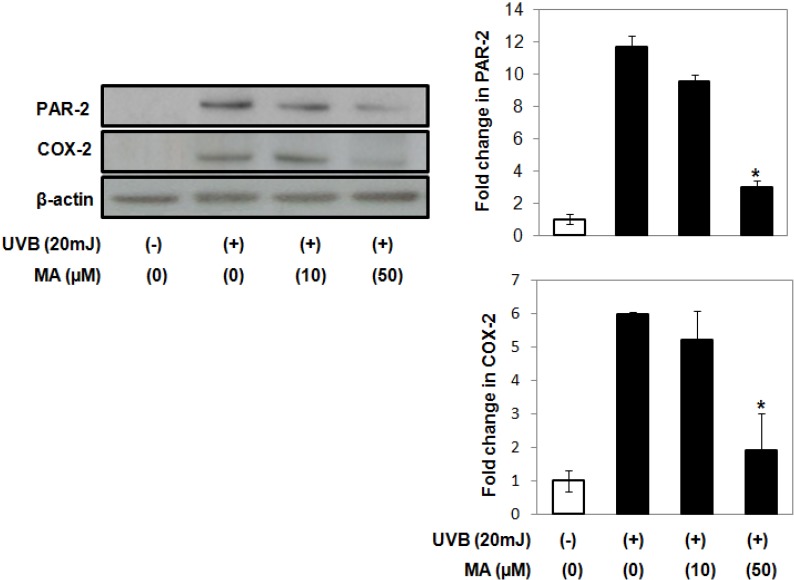
Effect of MA on COX-2 and PAR-2 expression in keratinocytes. Proteins were extracted from whole cell lysates of HaCaT keratinocytes. β-actin was used as a loading contril. The data shown are the mean ± S.D., n = 3; * *p* < 0.05 *vs.* UVB-irradiated control; MA: Madecassoside.

Exposure of keratinocytes to UV irradiation induces the expression of COX-2 and elevates the synthesis of PGs. In turn, COX-2 catalyzes the formation of proinflammatory prostaglandins (e.g., PGE_2_) from arachidonic acid [21]. PAR-2 has been linked to the upregulation of COX-2. We investigated whether UVB-induced increase of COX-2 and PAR-2 expression could be attenuated by MA in keratinocytes. UVB-induced expression of COX-2 and PAR-2 was inhibited by MA, suggesting that MA has anti-inflammatory effects on keratinocytes (Figure 3).

### 2.4. Effect of MA on Phogocytosis

**Figure 4:**
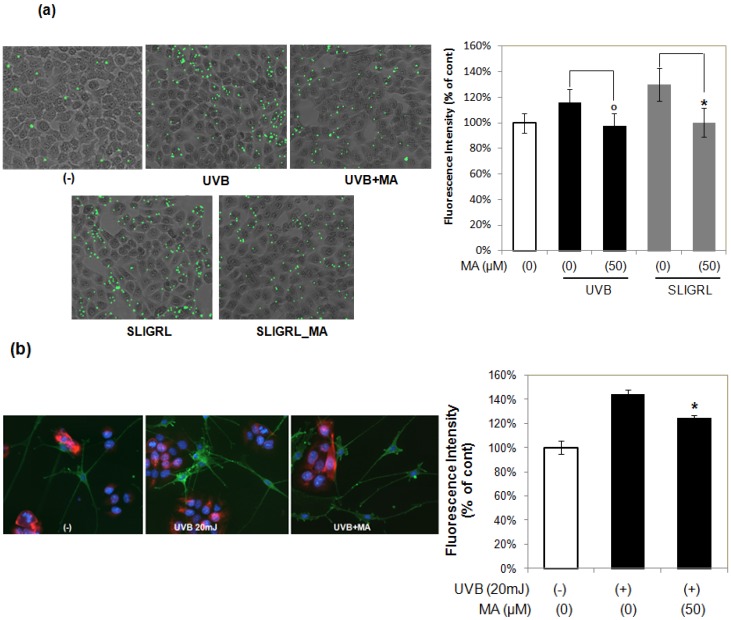
Effect of MA on melanosome phagocytosis. (**a**) Microsphere-based phagocytosis assay. ^o^* p* < 0.05 *vs.* UVB-irradiated control; * p < 0.05 *vs.* SLIGRL treated control. (**b**) Immunofluorescence analysis with MEL-5 co-culture system of melanocytes and keratinocytes. The data shown are the mean ± S.D., n = 3; * p < 0.05 *vs.* UVB-irradiated control; MA: Madecassoside.

PAR-2 is crucial in keratinocyte uptake of melanosomes. Activation of PAR-2 with Ser-Leu-Ile-Gly-Arg-Leu-NH(2) (SLIGRL), a known PAR-2 activating peptide, induces keratinocyte phagocytosis and increases skin pigmentation, indicating that PAR-2 regulates pigmentation by controlling phagocytosis of melanosomes. To assess the effect of MA on PAR-2-mediated keratinocyte phagocytosis, HaCaT keratinocytes were stimulated with SLIGRL (10 μM) and then incubated with fluorescently labeled microspheres (1 μm diameter). SLIGRL increased the phagocytic response (Figure 4a), but this effect was significantly attenuated by MA. To further elucidate the effects of MA on UVB-mediated phagocytosis, HaCaT keratinocytes were treated with UVB irradiation alone or with MA, and then incubated with fluorescently labeled microspheres. Consistent with its effect on phagocytosis in SLIGRL, MA treatment reduced the number of microspheres induced by UVB irradiation (Figure 4a). These results indicate that MA can inhibit UVB-induced phagocytosis by blocking PAR-2.

In addition, we used the *in vitro* co-culture system of melanocytes and keratinocytes to examine the involvement of MA in the distribution of melanosomes and dendritogenesis of melanocytes Keratinocytes/melanocytes co-cultures were treated with UVB irradiation alone or with MA. To indentify melanosomes, we used anti-MEL-5 antibody, which recognizes pg75 expressed in the melanosomes. The keratinocytes were stained with cytokeratin and the nuclei were stained with Hoechst, respectively. As shown in Figure 4b, the MEL-5 fluorescence intensity in MA-treated melanocytes appeared to decrease when compared to the UVB-irradiated control. UVB-irradiation stimulated dendritogenesis in melanocytes, whereas MA treatment inhibited these morphological changes, as assessed by fluorescence microscopy (Figure 4b). UVB-irradiation in keratinocytes stimulates PGE_2_ and PGF_2_α release, which acts as paracrine factor to stimulate melanin synthesis and melanocyte dendrite formation [5]. These results suggest that MA can inhibit UVB-induced increase of melanosomes and dendritogenesis by blocking PGE_2_ and PGF_2_α release from keratinocytes.

### 2.5. Effect of MA on Melanogenesis in Reconstructed Epidermis

**Figure 5:**
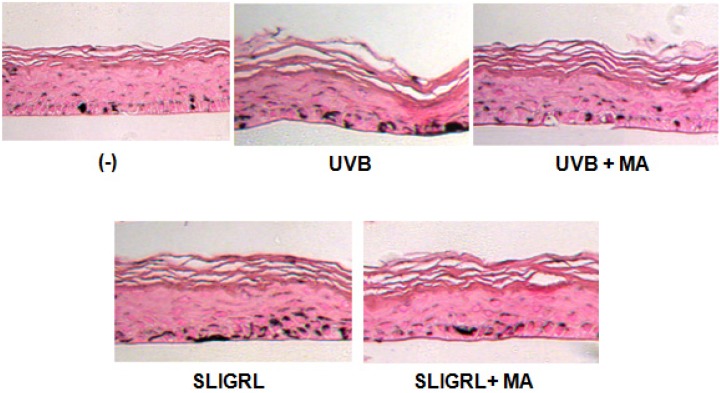
Effect of MA on reconstructed epidermis. Application of MA decreased melanin pigmentation induced by UVB radiation and SLIGRL in reconstructed epidermis. Histological sections after Fontana-Masson staining.

Several studies have reported that UVB irradiation enhances skin pigmentation by activating PAR-2 [9,22]. Melanoderm, a reconstructed epidermis containing functional melanocytes, was used to clarify the effects of MA in skin pigmentation. SLIGRL (10 μM) induced an increase in the melanin contents in the reconstructed epidermis compared with cultures grown without SLIGRL stimulation after 14 days of culture. In addition, UVB irradiation (100 mJ/cm^2^) induced an increase in the melanin contents. Melanin synthesis induced by SLIGRL was downregulated by MA treatment. Futhermore, UVB irradiation induced melanin synthesis was significantly suppressed by MA treatment (Figure 5).

### 2.6. Effect of MA on UVB-Induced Tannin

**Figure 6:**
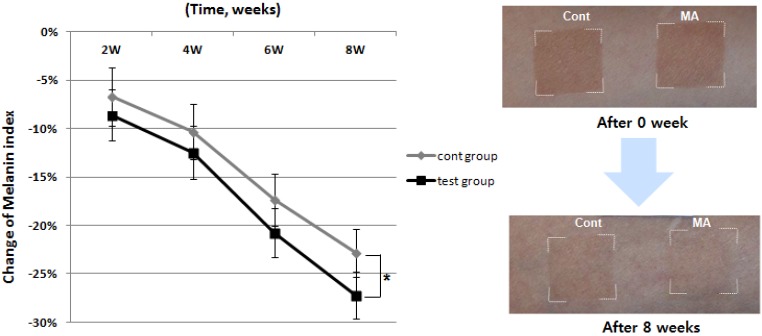
Effect of the MA on artificially tanned human skin *in vivo*. Analysis of melanin index by narrow band-spectral reflectance of light. Compared to the A group (control, cont), the melanin index was significantly decreased in the B group (MA, test) at 8 weeks (*p* < 0.05).

The whitening effect of MA was also evaluated *in vivo* using artificially-tanned skin. Twenty three healthy subjects (average age: 40.35 ± 5.43 years) with Fitzpatrick skin type III (sometimes mild burn, tan about average) and type IV (rarely burn, tan more than average with ease) who had no history of allergenic contact dermatitis participated in this study. The investigator fully explained the purpose and procedures of the study, schedule, compensation, and anticipated adverse reactions or side effects. Artificial tanning was induced at two sites on the forearm of all enrolled subjects by UV irradiation using a Multiport UV Solar Simulator (Solar Light Co., Glenside, PA, USA) at an exposure of 2 Minimal Erythema Dose (MED). The test material and the placebo were randomly allocated to the two tanned sites. The subjects applied the test material and the placebo twice daily (once in the morning and once in the evening) to the tanned sites for 8 weeks. All subjects were evaluated before treatment, and at 2, 4, 6, and 8 weeks after treatment. The melanin index was determined using a Mexameter® MX18 (C+K, Cologne, Germany). Compared to the control group, MA significantly reduced UV-induced melanin index at 8 weeks after topical application (Figure 6).

## 3. Experimental

### 3.1. Materials

Madecassoside was purchased from Santa Cruz Biotechnology (Santa Cruz, CA, USA). Monoclonal antibody to COX-2 and PAR-2 were purchased from Cell Signaling Technology (Beverly, MA, USA). Mel-5 antibody was purchased from SIGNET (Emeryville, CA, USA). SLIGRL was purchased from Peptron (Seoul, Korea). FluoSpheres® carboxylate modified fluorescent microspheres were purchased from Invitrogen (Carlsbad, CA, USA). Reconstructed human epidermis was purchased from MatTek (Ashland, MA, USA).

### 3.2. Cell and Cell Culture

Cultured normal human epidermal melanocyte cells obtained from Cascade Biologics (Portland, OR, USA) were maintained in Medium 154 (Cascade Biologicals) supplemented with 0.2% v/v bovine pituitary extract, 0.5% vol/vol fetal bovine serum, 5 μg/mL bovine insulin, 5 μg/mL bovine transferrin, 3 ng/mL basic fibroblast growth factor, 0.18 μg/mL hydrocortisone, 3 μg/mL heparin, and 10 ng/mL phorbol 12-myristate 13-acetate at 37 °C in a humidified atmosphere containing 95% air/5% CO_2_. HaCaT human keratinocyte cell line was obtained from CLS (Eppelheim, Germany) and was maintained in in Dulbecco’s modified Eagle’s medium (DMEM) supplemented with 10% fetal bovine serum (FBS) and 1% antibiotics.

### 3.3. UV Irradiation

For UVB exposure, when the cells were 70% confluent, the medium was removed and the cells were washed with phosphate buffered saline (PBS) and gently overlaid with DMEM devoid of phenol red (Biowhittaker, Lonza, Switzerland). UVB irradiation was conducted in DMEM devoid of phenol red using a Vilber-Lourmat device with an emission spectrum of 280–370 nm and a peak at 312 nm. The UV dose was measured with a model UV-340 UV light meter (Lutron, Coopersburg, PA, USA).

### 3.4. Co-Culture

Melanocytes and HaCaT keratinocytes were co-cultured with a slight modification of previously described previously methods [23,24]. For co-culture conditions, melanocytes were cultured (1 × 10^5^ cells/well) in a 12-well plate. HaCaT (5 × 10^4^) keratinocytes were seeded in 12-well Millicell Hanging Cell Culture Inserts (Millipore, Billerica, MA, USA) with 5 µm porous membrane separating the upper and lower chambers. The culture medium used before the establishment of the co-culture was the aforementioned standard growth medium for the two cell types. After 24 h, HaCaT keratinocytes in the hanging inserts were irradiated with the indicated doses of UVB and then changed with co-culture medium containing with the indicated MA concentration in the inserts above the melanocytes. Upon initiation of the co-cultures, the medium of upper and lower chamber was changed to serum-free keratinocyte growth medium (SF-KGM) with keratinocyte growth supplement (KGS; Invitrogen).

### 3.5. Melanin Assay

Melanocytes and HaCaT keratinocytes were co-cultured in the contact system in the presence or absence of UVB irradiation (20 mJ/cm^2^) using 12-well Millicell Hanging Cell Culture Inserts. After HaCaT keratinocytes in the upper chamber were irradiated with UVB, MA was added to indicated concentration above the melanocytes. After 4 days, the lower-chamber melanocytes were collected for the melanin assay. The assay was performed as previously described [25]. Briefly, cell pellets were solubilized in 1 N NaOH (60 °C) for 1 h. The color was analyzed at a spectrum of 475 nm. Melanin contents was determined by the absorbance/µg of protein in a cell extract.

### 3.6. Lipid Mediator of Inflammation Assay

HaCaT keratinocytes were irradiated with the indicated doses of UVB and incubated with the indicated madecassoside concentration for 24 h. PGE_2_ and PGF_2_α concentrations in the culture supernatant were measured using ELISA kits (R&D Systems, Minneapolis, MN, USA), according to the manufacturer’s instructions.

### 3.7. Western Blot Analysis

Western blot analysis was conducted by separating cell lysates via 10% SDS-PAGE. The gels were blotted overnight on polyvinylidene difluoride membranes, after which they were exposed to the appropriate antibodies (anti-COX2, anti-PAR-2). The proteins were then visualized using enhanced chemiluminescence, ECL western blotting system (Amersham Biosciences, Piscataway, NJ, USA) with horseradish peroxidase-conjugated antirabbit or anti-mouse as the secondary antibody. The results were confirmed by three independent experiments.

### 3.8. Phagocytosis Assay

Microsphere-based phagocytosis assay HaCaT keratinocytes were seeded in 24-well plates, incubated overnight at 37 °C, and treated with SLIGRL (10 μM) or irradiated with UVB (20 mJ/cm^2^) in the presence or absence of MA. After 24 h, cells were then serum-starved for 6 h. FluoSpheres^®^ carboxylatemodified yellow-green fluorescent microspheres (1 μm diameter; Invitrogen) were prepared 24 h before use according to the manufacturer’s instructions. Cells were incubated with microspheres (100/cell) at 37 °C for 4 h. The microspheres were aspirated and cells were incubated with 1 mL FBS at 37 °C for 15 min. After extensive washing, images of the ingested beads were taken. Each experiment was performed in duplicate and repeated at least three times.

### 3.9. Immunofluorescence Staining

Melanocytes and HaCaT keratinocytes were subcultured onto vitrogen-coated 8-well chamber slides (Nalge Nunc International, Naperville, IL, USA) and maintained in a 1:1 mixture of melanocyte culture medium (Medium 154s) and HaCaT cells culture medium (DMEM containing 10% FBS). Cell monolayers were then fixed in 2.5% glutaraldehyde and 2% paraformaldehyde in 0.1 M phosphate buffer (pH 7.4), after which they were permeabilized in 0.1% Triton-X-100 in stabilization buffer (PBS, 100 mM MgCl_2_, 1 mM CaCl_2_) for 10 min. Next, the non-specific binding of antibodies was blocked by incubating the slides in 10% normal goat serum. Primary antibodies were then applied overnight at 4 °C, followed by incubation with appropriate fluorescein-conjugated secondary antibodies for 2 h at room temperature. Hoechst 33342 (Invitrogen) was used to stain the nuclei. Images were captured with a Spot digital camera and post-processed using Olympus DP manager (Olympus, Tokyo, Japan).

### 3.10. Histochemistry of Reconstructed Epidermis

Reconstructed human epidermis (MatTek, Ashland, MA, USA) consisted of normal human-derived epidermal keratinocytes and normal human epidermal melanocytes that had been cultured to form a multilayered, highly differentiated model. Reconstructed epidermis was incubated in medium containing 50 μM MA for 2 weeks. The chemical was added in the lower well of the reconstructed epidermis system, and then penetrated through a membrane to reach the basal cells. The medium containing MA was replaced every other day. The epidermis was then fixed with 4% formalin in PBS followed by Fontana–Masson staining to visualize melanin pigments. Images were analyzed using Image-Pro Plus version 4.1.

### 3.11. Clinical Study of MA on UV-Induced Tanning Skin

Twenty three healthy subjects (average age: 40.35 ± 5.43 years) with Fitzpatrick skin type III (sometimes mild burn, tan about average) or IV (rarely burn, tan more than average) participated in this study. Fitzpatrick skin type classifies skin reaction to UV irradiation. The MED is defined as the lowest time interval or dosage of UV light irradiation sufficient to produce a minimal, perceptible erythema on unprotected skin and essential for rational treatment with UV light to induce artificial pigmentation. To determine the MED of each subjects, each forearm was irradiated with a test dose ladder of 16–48 mJ/cm^2^ using Multiport UV Solar Simulator (Solar Light Co., Glenside, PA, USA). MED and erythema index were read after 24 h. After finding out MED of each subject, artificial tanning was induced at two sites on the forearm by 2 MED. Skin conditions of all subjects were evaluated before treatment and at 2, 4, 6, and 8 weeks after treatment. Skin whitening was evaluated by the melanin index. The products (test serum: MA 0.05%, placebo: non-treated MA) were applied at different intervals on each test sites (A group: placebo used after pigmentation, B group: test serum used after pigmentation) twice daily (once in the morning and once in the evening) for 8 weeks. This study was approved by the ethics committee of the DERMAPRO/Skin Research Center (Seoul, Korea). Subjects gave written informed consent.

### 3.12. Statistical Analysis

The statistical significance of the data was determined by a one-way or two-way ANOVA method. The melanin index of clinical study was statistically analyzed using RM ANOVA and ANOVA of SPSS 11.5 package program. A *p* < 0.05 was considered to be significant.

## 4. Conclusions

MA significantly inhibits UV-induced melanin synthesis and melanosome transfer in a co-culture system of keratinocytes and melanocytes by supressing PAR-2 expression and its signaling pathway involving COX-2, PGE_2_, and PGF_2_α in keratinocytes. MA may be an effective inhibitor of hyperpigmentation caused by UV irradiation.

## Supplementary Materials

Supplementary materials can be accessed at: http://www.mdpi.com/1420-3049/18/12/15724/s1.
