# Supplementary material for: Madecassoside Inhibits Melanin Synthesis by Blocking Ultraviolet-Induced Inflammation
Source: Molecules. 2013 Dec 16;18(12):15724–36. doi: 10.3390/molecules181215724 (PMC6290557; doi:10.3390/molecules181215724)

Supplement Information

Figure S1. Effects of MA on melanin synthesis in single melanocyte model.

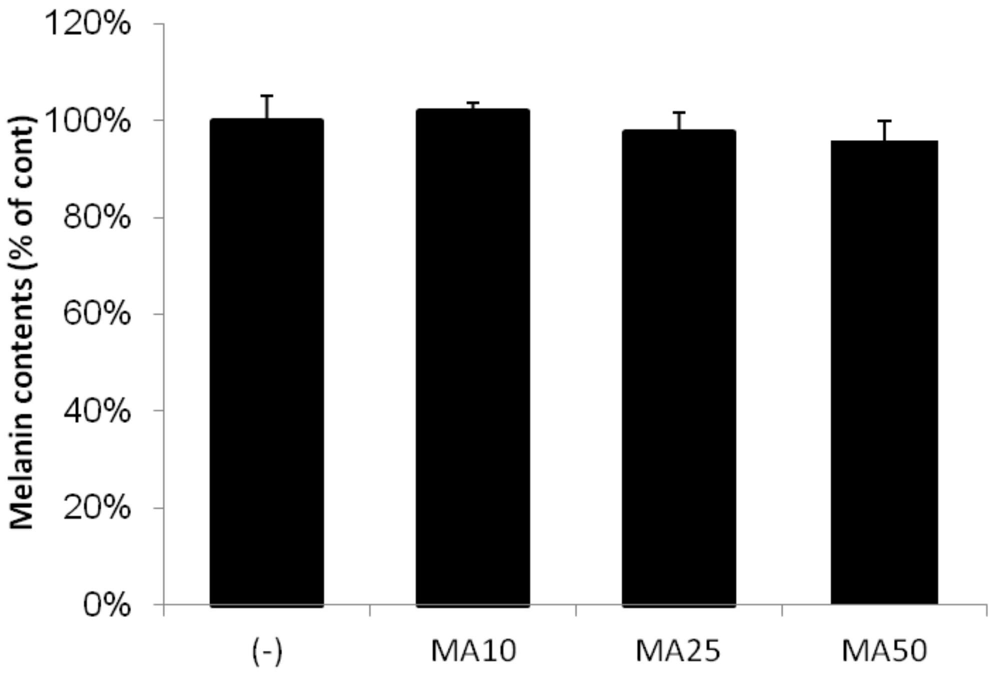

Figure S2. Full-length images of immunoblots.

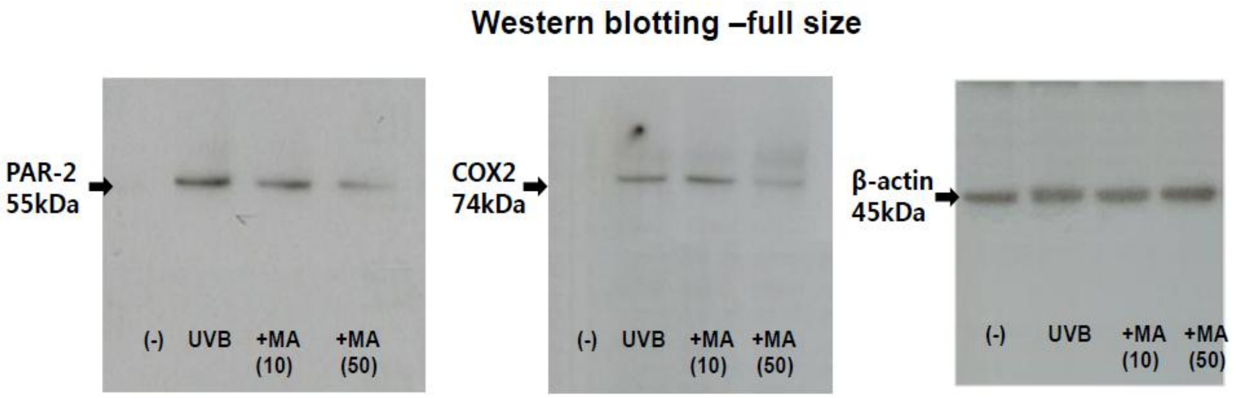

Supplement: Supplementary file 1 [file molecules-18-15724-s002.pdf]
